# Effects of arsenic exposure on DNA methylation in cord blood samples from newborn babies and in a human lymphoblast cell line

**DOI:** 10.1186/1476-069X-11-31

**Published:** 2012-05-02

**Authors:** Ponpat Intarasunanont, Panida Navasumrit, Somchamai Waraprasit, Krittinee Chaisatra, William A Suk, Chulabhorn Mahidol, Mathuros Ruchirawat

**Affiliations:** 1 Laboratory of Environmental Toxicology, Chulabhorn Research Institute, Bangkok, 10210, Thailand; 2 Inter-University Post Graduate Program in Environmental Toxicology, Technology and Management of the Chulabhorn Research Institute, Asian Institute of Technology and Mahidol University, Center of Excellence on Environmental Health and Toxicology, CHE, Ministry of Education, Bangkok, Thailand; 3 Center for Risk and Integrated Sciences, National Institute of Environmental Health Sciences, Research Triangle Park, North Carolina, USA; 4 Laboratory of Chemical Carcinogenesis, Chulabhorn Research Institute, Bangkok, 10210, Thailand; 5 Department of Pharmacology, Faculty of Science, Mahidol University, Phayathai, Bangkok, 10400, Thailand

**Keywords:** Arsenic, Exposure *in utero*, Global DNA methylation, p53 promoter methylation, Lymphoblast cell line, Cord blood lymphocyte

## Abstract

****Background**:**

Accumulating evidence indicates that *in utero* exposure to arsenic is associated with congenital defects and long-term disease consequences including cancers. Recent studies suggest that arsenic carcinogenesis results from epigenetic changes, particularly in DNA methylation. This study aimed to investigate DNA methylation changes as a result of arsenic exposure *in utero* and *in vitro*.

****Methods**:**

For the exposure *in utero* study, a total of seventy-one newborns (fifty-five arsenic-exposed and sixteen unexposed newborns) were recruited. Arsenic concentrations in the drinking water were measured, and exposure in newborns was assessed by measurement of arsenic concentrations in cord blood, nails and hair by Inductively Coupled Plasma Mass Spectrometry (ICP-MS). In the *in vitro* study, human lymphoblasts were treated with arsenite at 0-100 μM for two, four and eight hours (short-term) and at 0, 0.5 and 1.0 μM for eight-weeks period (long-term). DNA methylation was analyzed in cord blood lymphocytes and lymphoblasts treated with arsenite *in vitro*. Global DNA methylation was determined as LINE-1 methylation using combined bisulfite restriction analysis **(**COBRA) and total 5-methyldeoxycytidine (5MedC) content which was determined by HPLC-MS/MS. Methylation of p53 was determined at the promoter region using methylation-specific restriction endonuclease digestion with MspI and HpaII.

****Results**:**

Results showed that arsenic-exposed newborns had significantly higher levels of arsenic in cord blood, fingernails, toenails and hair than those of the unexposed subjects and a slight increase in promoter methylation of p53 in cord blood lymphocytes which significantly correlated with arsenic accumulation in nails (p < 0.05) was observed, while LINE-1 methylation was unchanged. Short-term *in vitro* arsenite treatment in lymphoblastoid cells clearly demonstrated a significant global hypomethylation, determined as reduction in LINE-1 methylation and total 5-MedC content, and p53 hypermethylation (p < 0.05). However, a slight LINE-1 hypomethylation and transient p53 promoter hypermethylation were observed following long-term *in vitro* treatment.

****Conclusions**:**

This study provides an important finding that *in utero* arsenic exposure affects DNA methylation, particularly at the p53 promoter region, which may be linked to the mechanism of arsenic carcinogenesis and the observed increased incidence of cancer later in life.

## **Background**

Exposure to arsenic is a major health concern worldwide. Arsenic is an environmental pollutant that has been classified as a human carcinogen by the International Agency for Research on Cancer [1] and the US Environmental Protection Agency [2]. Epidemiological studies have shown that chronic exposure to arsenic in drinking water is associated with increased risk of various diseases, including cancers [3]. A number of studies have reported that possible mechanisms of arsenic carcinogenesis include both genotoxic and non-genotoxic mechanisms [4]. Genotoxic effects of arsenic involve chromosome aberrations at toxic concentrations and aneuploidy as well as delayed mutagenesis at non-toxic concentrations [5]. Among genotoxic effects of arsenic in humans, chromosome aberrations and micronuclei in different cell types have been found to be significantly increased [6]. However, a growing body of evidence suggests that arsenic exerts its carcinogenicity through epigenetic mechanisms by affecting multiple cellular pathways, including expression of growth factors, suppression of cell cycle check points, promotion of and resistance to apoptosis, inhibition of DNA repair, and alterations in DNA methylation [7,8].

Epigenetic dysregulation of gene expression as a result of DNA methylation changes plays an important role in cancer etiology [9,10]. Global hypomethylation and aberrant promoter methylation of tumor suppressor genes are frequently found in cancer tissues [11,12]. The epigenetic effects of arsenic have been extensively studied, particularly on DNA methylation alterations. Changes in gene methylation status mediated by arsenic have been proposed to activate oncogene expression or silence tumor suppressor genes, leading to long-term changes in the activity of genes controlling cell transformation. Arsenic exposure has been reported to cause a significant reduction in global DNA methylation associated with malignant transformation in rat liver epithelial cells [13] and to induce hypermethylation within the promoter region of the tumor suppressor *p53* gene in human lung adenocarcinoma A549 cells [14]. In addition, Chanda et al [15] reported that chronic arsenic exposure is associated with the promoter hypermethylation of p53 and p16 in lung adenoma in arsenic-exposed residents who were chronically exposed to arsenic-contaminated drinking water. Recently, it has been reported that arsenic pollution from coal-burning caused p53 promoter hypermethylation in one hundred and twelve Chinese patients with arsenism [16]. The hypermethylation of p53 at the promoter region was related with the mutation of the *p53* gene (exon 5), and hypomethylation of p53 at exon 5 was also related with its mutation. The data implicated that arsenic affects both DNA methylation and mutation of *p53*. The tumor suppressor gene, *p53*, is a key factor in DNA damage-signalling pathways, and p53 hypomethylation is associated with DNA strand breaks and chromosome instability [17].

Arsenic contamination affects millions of people who are exposed to drinking water at arsenic levels that exceed the World Health Organization (WHO) safety standard of 10 μg/L. Among arsenic-exposed populations, children are considered to be a vulnerable group to arsenic exposure. Epidemiological evidence links *in utero* and early-life human exposures to arsenic with increased risk of cancer mortality during early adulthood [18-20], as well as with developmental and long-term health consequences, including pulmonary and cardiovasuclar diseases, fetal loss and birth defects [21]. In an experimental animal study, *in utero* exposure to inorganic arsenic resulted in a variety of tumors in the off-spring when they reached adulthood [22-24]. Additionally, it has been proposed that gestational disruption of normal epigenetic programming by arsenic may lead to aberrant gene expression, which in turn influences fetal development and disease risk later in life [25].

In Thailand, one of the areas heavily contaminated with arsenic is Ron Pibul District of Nakhon Sri Thammarat Province. This area was the site of tin mining activities for almost a century. Although most of the mines ceased operation in the late 1980s, the release of arsenopyrite (FeAsS) from the tin ore caused extensive arsenic contamination in many areas of Ron Pibul District. Human health problems associated with arsenic toxicity in Ron Pibul District were first recognized in 1987, with the risk of arsenic poisoning in the population due to drinking and using contaminated water daily. Reported levels of arsenic in these areas range from <0.26 to 1000 μg/L [26,27]. Recently, we reported for the first time that prenatal arsenic exposure in a human population living in this district, resulted in alarming gene expression changes in the newborns of exposed mothers. Altered expression of these arsenic-associated genes shows a striking dose response relationship to prenatal arsenic exposure, and a biological network analysis showed that the arsenic-associated transcripts could modulate numerous biological pathways, including apoptosis, cell signaling, inflammation and stress response, and ultimately affect health status [28].

Further to the aforementioned studies on gene expression, the present study was conducted in the cord blood samples from the same studied groups to examine the effects of arsenic exposure *in utero* on DNA methylation, focusing on global DNA methylation and p53 promoter methylation in cord blood lymphocytes of newborn babies. In parallel with the *in utero* exposure study, *in vitro* studies using a human lymphoblast cell line exposed to arsenic were also carried out.

## **Methods**

### **Study locations and subjects**

The study was conducted in arsenic contaminated areas in the Ron Pibul District, Nakhon Sri Thammarat Province, which is located in the southern peninsula of Thailand. Ron Pibul District covers an area of approximately five hundred square kilometers and has been the site of tin mining activities for almost a century. Although most of the mines ceased operation in the late 1980s, the release of arsenopyrite (FeAsS) from the tin ore has resulted in arsenic contamination in many areas. Five villages in the Ron Pibul District were selected for this study as they had been classified as high-level arsenic contaminated areas and arsenicosis. Arsenicosis has not been reported in central part of Thailand, where arsenic concentrations in water and soil have been determined to be very low [29]. Thus, unexposed subjects were selected from those living in the unexposed area and came to a hospital in Bangkok for delivery. Arsenic levels in fingernails of pregnant women confirmed exposed and unexposed status.

A total of seventy-one newborns, consisting of fifty-five newborns from mothers living in the Ron Pibul District and sixteen newborns from unexposed mothers, were the subjects in this study. All mothers of the newborns in this study were healthy, non-smokers, had no complications during pregnancy and underwent vaginal childbirth without birth stimulation or anesthesia. The mothers from both groups were age, educational level, and socioeconomically matched. Questionnaires were administered to all participants to obtain personal information regarding residential history, health history and potential confounding factors, birth and pregnancy information (number of births, abortions or complications), use of community drinking water and well water, as well as water and food consumption habits. Maternal urinary cotinine level was checked consequently and showed to be in the range of 0-0.43 μg/mmol creatinine, lower than reference background level of 28 μg/mmol creatinine [30]. Cord blood samples were collected from January 2004 to December 2005 in the Ron Pibul Hospital (Ron Pibul District) and the Rajvithi Hospital (Bangkok). This study was conducted according to the recommendations of the Declaration of Helsinki (World Medical Association 1989) for international health research. All pregnant mothers gave written informed consent to participate in this study.

### **Measurement of arsenic concentration in water samples**

Water samples were collected in 500 mL plastic bottles and HNO_3_ was added to the water samples, which were stored at 5°C until analysis. The collected samples were filtered through 0.45 μm filter membranes prior to analysis of the dissolved arsenic concentration using ICP-MS.

### **ICP-MS analysis of arsenic concentration**

Arsenic concentrations in the samples were analyzed using an Octopole ICP-MS. The sample introduction system for ICP MS consists of a Babington-type nebulizer and spray chamber cooled by a Peltier system. Arsenic was monitored at *m/z* = 75, the mass of the only arsenic isotope. Instrumentation parameters were Rf power = 1500 W, Carrier gas = Ar, flow rate = 0.8 L/minute, makeup gas = 0.29 L/minute, nebulizer pump = 0.1 rps, measurement mode = peak area of ^75^As, Integration time = 0.1 second, and point per peak = 1.

### **Analysis of arsenic concentration in cord blood**

Analysis of arsenic concentrations in cord blood was carried out according to a previously reported procedure, with modification [31]. Cord blood samples were drawn from the cubical vein into sterilized tubes containing heparin (2 units /mL). The cord blood samples were stored at -70 ^o^C until analysis. A 300 μL aliquot of blood was digested with 1 mL suprapure nitric acid using a microwave oven (Milestone ETHOS) with a constant power (900 watts) and programmable temperature at 110 ^o^C, 180 ^o^C and 200 ^o^C for 3, 7 and 5 minutes, respectively. The digested samples were analyzed for total arsenic concentrations by ICP-MS, with the analysis parameters as previously described.

### **Analysis of arsenic accumulation in hair and nails**

Analysis of arsenic accumulation in hair and nails was carried out according to a previously reported procedure, with modification [32,33]. Fingernails and toenails were clipped and hair samples were cut. The samples were kept in a zip-lock bag at room temperature until analysis. The samples both nails and hair (20-30 mg) were transferred to a polyethylene vial and sequentially washed by adding 2 mL acetone and sonicating in an ultrasonic bath for twenty minutes, followed by sonicating the samples with 1 % Triton X-100 for twenty minutes, then washing five times with deionized water and dried. This washing procedure removes all external contamination without extracting metals from the nails. The samples were dried and mixed with 1 mL concentrated HNO_3_. Subsequently, samples were microwave digested until the solution was clear. The digested samples were diluted to 5 mL with ultrapure water and subjected to ICP-MS analysis of arsenic concentration as previously described.

### **Isolation of cord blood lymphocytes**

Human cord blood was collected in 50 mL tubes containing heparin and kept on ice before lymphocyte separation. The lymphocytes were isolated by buoyant density using Ficoll-Paque plus (Amersham). Blood sample was mixed with one volume of RPMI 1640 medium and carefully layered in a 50 mL centrifuge tube containing 12 mL Ficoll-Paque. After centrifugation at 2500 rpm for thirty minutes at 18-20 °C, the upper layer was removed and the interface layer collected into a new tube, washed with two volumes of PBS and centrifuged at 1200 rpm for ten minutes at 18-20 °C. The supernatant was then removed and the lymphocyte pellet was resuspended with PBS. The number of lymphocytes was determined using a hemacytometer. The lymphocytes were stored at 5 x 10^6^ cells /mL in cold freezing medium. Cell suspension (2 mL) was transferred to a cryovial, submerged in a cryo-freezing container and immediately placed in a -80 °C freezer until analysis.

### **Cell culture and NaAsO**_**2**_**treatment**

A lymphoblast cell line (RPMI1788), established from human peripheral blood lymphocytes (profile# TKG0464), was obtained from the ATCC (#CCL-156). Cord blood lymphocytes were obtained from participating subjects (newborns). All cells were cultured in RPMI1640 medium containing 20 % FBS at 37 °C, 5 % CO_2_, free from antibiotics for twenty-four hours prior to treatment.

The inorganic trivalent arsenic compound (NaAsO_2_) was dissolved in the culture medium immediately prior to use. 1 M of NaAsO_2_ solution was diluted in order to make the final concentration in cell culture medium; 0, 10, 20, 50 and 100 μM for the short-term experiment, and 0.0, 0.5, 1.0 μM for the long-term experiment. There was duplication of samples at each concentration. Cells were incubated at 37 °C, 5 % CO_2_ for twenty-four hours prior to analysis.

### **Determination of DNA methylation**

#### **DNA isolation**

DNA was isolated from cord blood lymphocytes or lymphoblasts using DNeasy® blood & tissue kits (Qiagen). A fraction of 5 x 10^6^ cells was centrifuged and resuspended in 200 μL PBS. 20 μL protienase K and 4 μL RNase A (100 mg/mL) were added and incubated for two minutes at room temperature, lysed in 200 μL buffer AL and incubated at 56°C for ten minutes. Ethanol (200 μL) was added, mixed and transferred to a DNeasy mini spin column, which was then centrifuged at 8,000 rpm for one minute. The column was sequentially washed with AW1 and AW2 buffer. The bound DNA was eluted with 80 μL buffer AE, incubated at room temperature for one minute and centrifuged at 8,000 rpm for one minute. The purity and concentration of total DNA were determined by the ratio of absorbance at 260/280 and 260/230 nm using a Nanodrop ND-1000.

#### **Analysis of global LINE-1 methylation using combined bisulfite restriction analysis (COBRA)**

Analysis of global LINE-1 methylation using combined bisulfite restriction analysis (COBRA) was carried out according to previously reported method, with modification [34]. DNA was isolated using a Qiagen DNA isolation kit, then COBRA using the bisulfite conversion kit (Qiagen) was performed. This method is widely used and accepted in DNA methylation studies in a variety of tissues [35-37]. A fraction of DNA (2 μg) was treated with bisulfite using a EpiTect® Bisulfite kit (Qiagen), which converted all cytosines to uracils, except methylated cytosines. The bisulfite DNA conversion was performed using a thermal cycler according to instructions in the EpiTect® Bisulfite kit manual. The bisulfite product was amplified with the specific long interspersed nucleotide elements (LINE-1 repetitive element (413 bp)), a surrogate marker of global DNA methylation, by PCR using the following primers and conditions: Primers F: 5’-TTGAGTTGTGGTGGGTTTTATTTAG-3’ R: 5’-TCATCTCACTAAAAAATACCAAACA-3’ PCR condition: (35 cycles) was denaturation at 95 °C for 30 seconds, annealing at 52 °C for 30 seconds, extension at 72 °C for 30 seconds. The PCR products (413 bp) were digested with HinfI restriction enzyme (Fermentas), which only recognizes and cuts repetitive elements that are originally methylated. The digested PCR products were analyzed and quantified by capillary electrophoresis using an Agilent Bioanalyzer. A sample of each digested PCR product (1 μL) was loaded onto a DNA chip according to the Agilent DNA 1000 kit protocol and analyzed by the Bioanalyzer 2100 software. Size of unmethylated DNA fragment was 413 bp and the methylated DNA fragments were 247, 166, and 128 bp. LINE-1 methylation of each sample was determined as percentage of methylated cytosine in relation to total cytosine.

#### **Measurement of 5-methyl-2’-deoxycytidine (5MedC) content using HPLC-ESI-MS/MS**

Genomic DNA was isolated from cord blood by using the QIAamp DNA blood Maxi kit according to the recommendations of the manufacturer. DNA was enzymatically digested to the deoxynucleoside. Briefly, 25 μg of DNA sample was incubated with 5U of nuclease P1 at 37°C for ten minutes. Subsequently, 10U of alkaline phosphatase was added and the mixture was incubated at 37°C for two hours. Following digestion, the hydrolysate was filtered through a 0.22-mm syringe filter before analysis. A fraction of 10 μl of DNA hydrolysate was subjected to analyze 5-MedC and dC by HPLC (Agilent 1200 series) equipped with a triple quadrupole mass spectrometer (Agilent 6410 Triple Quad LC/MS).

For HPLC-MS/MS analysis, the HPLC was connected to a Guanine adduct column (3.0 x 150 mm, ESA Inc., USA) with the column temperature set to 25°C. A flow rate of 0.5 mL/min was used for the mobile phase of methanol and 0.1 % formic acid. The MS/MS system was operated *via* an electrospray source with a positive ion mode. The capillary voltage was 4 kV. Nitrogen gas was used as a nebulizer gas by setting a flow rate of 9 L/minute and a pressure of 40 psi and a temperature at 275°C. The product ion transition of analytes was monitored in the MRM mode at m/z 242.1 → m/z 126.3 for 5-MedC and m/z 228.2 → m/z 112.2 for dC. The results of 5-MedC content was determined as total 5-MedC relative to dC.

#### **Determination of p53 promoter methylation**

p53 promoter methylation was determined according to a previously reported method, with modification [14]. DNA was isolated from cells using Qiagen DNA isolation kit. Methylation-specific restriction endonuclease digestion was performed using MspI & HpaII (Promega). The promoter region of the p53 gene contains 2 CCGG sites beginning at bases 703 and 883 (Gen Bank accession no: X 54156). The HpaII restriction enzyme cleaves CCGG sequences, which are not methylated at the internal or external cytosine. MspI is the isoschizomer of HpaII, and cleaves the CCGG sequence irrespective of its methylation status. Therefore, MspI cleaves both methylated and unmethylated cytosine while HpaII cleaves only unmethylated cytosine. MspI was used as a control for HpaII digestion. PCR amplification of the sequence in region (638-978 bp) cannot be performed if one of the CCGG sequences has been cleaved. DNA methylated at both the CCGG sequences of the p53 promoter is resistant to HpaII digestion and can be amplified by PCR. Genomic DNA (300 ng) was digested with two units of HpaII or MspI at 37 °C for five hours. Enzymes were inactivated at 95 °C for ten minutes. The HpaII and MspI-digested DNA were amplified using p53 promoter region primers (F: 5’-AGGGAATTCGGCACCAGGTCGGGGAGA-3’ R: 5’-AGGATCGATGGACTCATCAAGTTCAGT-3’) for 31 cycles giving rise to a 341 bp PCR product. PCR condition was at 94 °C for one minute, 58 °C for seventy seconds, 72 °C for three minutes. The PCR products (341 bp) were cleaned using a Qiagen MinElute purification kit prior to analysis with Bioanalyzer (Agilent). A sample of each amplified DNA (1 μl) was loaded onto a DNA chip according to the Agilent DNA 1000 kit protocol and assayed using the Bioanalyzer 2100 software. The methylation of p53 was calculated as the ratio of HpaII: MspI product.

### **Statistical analysis**

The Mann-Whitney U Test was used to determine statistical differences of the test parameters. The correlations between p53 promoter methylation and arsenic accumulation in nails were determined by Pearson correlation coefficient. A p-value less than 0.05 was considered to represent statistical significance.

## **Results**

### **Arsenic concentrations in various sources of water**

Arsenic concentrations in water samples from various sources including bottled water, local supply tap water, mountain pipe water, well water and rainwater in the contaminated (Ron Pibul District) and reference (control) sites are shown in Tables 1 and 2. High levels of arsenic contamination were found in well water in the contaminated sites, where the concentrations were varied from 1-1,475 μg/L. Varying concentrations of arsenic were also found in other sources of water, including mountain pipe water and local supply of tap water, but the mean levels were approximately 12-fold lower than that in the well water. In this site, the arsenic concentrations detected in rainwater and bottled water were 1.01 and 4.54 μg/L, respectively, which are under the World Health Organization (WHO) guideline value (<10 μg/L) for arsenic in drinking water. In the control site, the arsenic concentrations in bottled water and tap water were much lower than those from the contaminated site. Arsenic levels in drinking and non-drinking water were approximately 46-fold (p < 0.05) and 78-fold (p < 0.05) higher, respectively, in the samples from arsenic-contaminated site (Table 2).

**Table 1:** Arsenic concentrations in various sources of water from the study locations

**Sources of water**	**Arsenic concentrations (μg/L)**	
	**Study locations**	
	**Arsenic-contaminated site**	**Control site**
Well water	298.18 ± 129.60 ^a^	nd
(n = 13)	87.36 (1.11-1,475.00)^b^	
Mountain pipe water	25.54 ± 9.02	nd
(n = 10)	6.86 (1.59-75.05)	
Local tap water	24.22 ± 17.23	0.22 ± 0.11
(n = 12)	4.86 (0.53-147.60)	nd (nd-1.09)
Rain water	1.01 ± 0.20	nd
(n = 23)	0.65 (0.17-2.99)	
Bottled water	4.54 ± 0.43	0.20 ± 0.10
(n = 6)	4.20 (3.45-5.85)	0.06 (nd-1.20)

The values are expressed as the mean ± SE (^a^) and median (min - max) (^b^).

nd : non detectable.

**Table 2:** Arsenic concentration in drinking and non-drinking water samples

**Study locations**	**Arsenic concentration (μg/L)**	
	**Drinking water**	**Non-drinking water**
Arsenic-contaminated site	8.38 ± 2.49 ^a,*^	78.05 ± 20.92^*^
(n =46)	0.86 (0.17-61.63)^b^	16.54 (1.14-518.69)
Control site	0.18 ± 0.07	0.99 ± 0.04
(n = 18)	0.04 (nd-0.98)	1.10 (nd-1.05)

The values are expressed as the mean ± SE (^a^) and median (min - max) (^b^).

nd : non detectable.

^*^ : statistically significant difference from controls at p < 0.05.

### **Arsenic concentrations in biological samples from newborns**

Levels of arsenic accumulation in biological samples from newborns are shown in Table 3. When compared to samples from the newborns from the control site, arsenic accumulation in newborns from exposed mothers living in the contaminated site was significantly greater by approximately 3-, 12-, 24-, and 5-fold in cord blood, toenails, fingernails and hair, respectively, thus confirming exposure in these babies.

**Table 3:** Arsenic concentrations in biological samples from newborns

**Groups**	**Arsenic concentrations**			
	**Cord blood (μg/g)**	**Toenails (μg/g)**	**Fingernails (μg/g)**	**Hair (μg/g)**
Exposed	5.79 ± 0.5 ^a,*^	1.52 ± 0.38^***^	1.91 ± 0.38^**^	0.05 ± 0.01^***^
(n = 55)	6.27 (1.31-10.37)^b^	0.76 (nd-8.23)	1.45 (nd-9.08)	0.031 (nd-0.38)
Control	1.97 ± 0.64	0.12 ± 0.04	0.08 ± 0.05	0.01 ± 0.003
(n = 16)	1.25 (0.51-8.31)	0.03 (nd-0.59)	0.06 (nd-0.41)	nd (nd-0.06)

The values are expressed as the mean ± SE (^a^) and median (min - max) (^b^).

nd : non detectable.

^*^,^**^, ^***^ : statistically significant difference from controls at p < 0.05, 0.01 and 0.001, respctively.

### **Global DNA methylation and p53 promoter methylation in human cord blood lymphocytes as a result of arsenic exposure*in utero***

**Figure 1:**
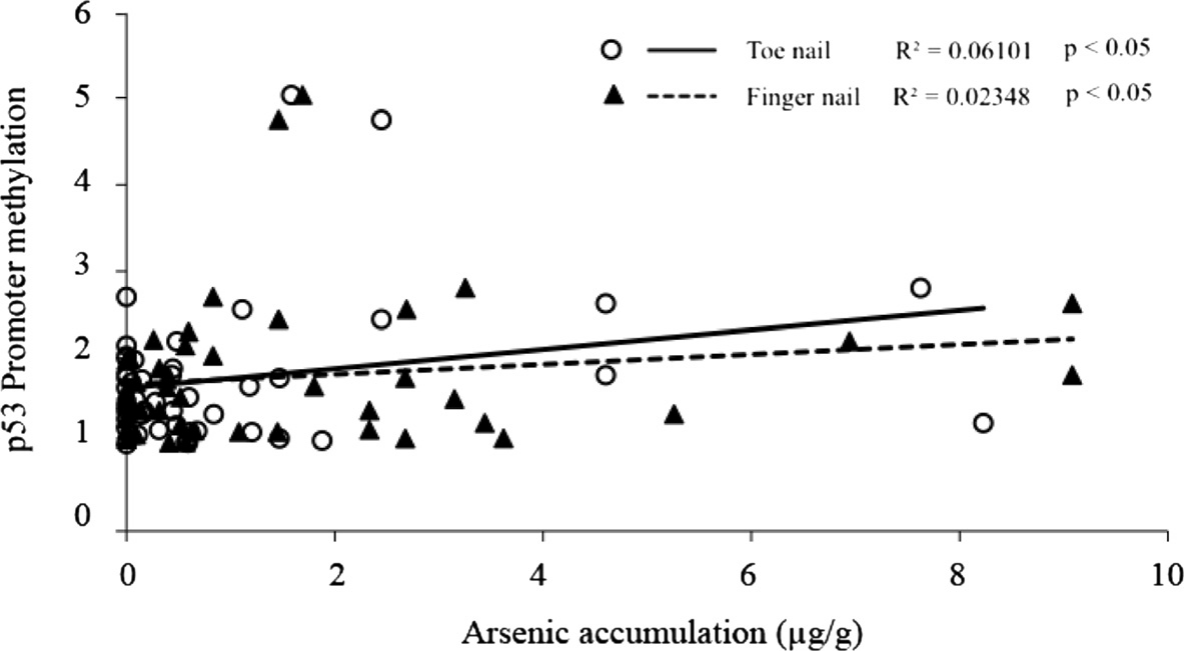
**Scatter plot of non parametric correlation between the level of p53 promoter methylation and arsenic accumulation in toenails or fingernails from both exposed and unexposed newborns.** R^2^ represent correlation coefficient and p represent p-value of the correlation.

Global DNA methylation and p53 promoter methylation were determined in cord blood lymphocytes from exposed (n = 55) and control subjects (n = 16). As shown in Table 4, the difference in levels of global LINE-1 DNA methylation expressed as percentage of methylated cytosine in relation to total cytosine between the exposed- and control groups, was not statistically significant. Methylation of the p53 promotor region was slightly higher in the arsenic-exposed group, but a significant correlation between the level of methylation and accumulation of arsenic in toenails or fingernails was observed (p < 0.05) (Figure 1).

**Table 4:** Global DNA methylation and p53 promoter methylation in newborns exposed to arsenic and control groups

**Groups**	**DNA methylation**	
	**LINE-1**	**p53 promoter**
	**(% Methylation)**	**(Methylated cytosine: cytosine)**
Exposed	83.01 ± 0.40^a^	1.77 ± 0.11
(n = 55)	83.47 (73.02 - 89.15)^b^	1.66 (1.03 - 5.03)
Control	84.44 ± 0.64	1.53 ± 0.09
(n = 16)	84.87 (79.34 - 88.33)	1.48 (1.01 - 2.19)

The values are expressed as the mean ± SE (^a^) and median (min - max) (^b^).

### **Effects of*in vitro*arsenic exposure on global DNA and p53 promotor methylation in human lymphoblasts**

**Figure 2:**
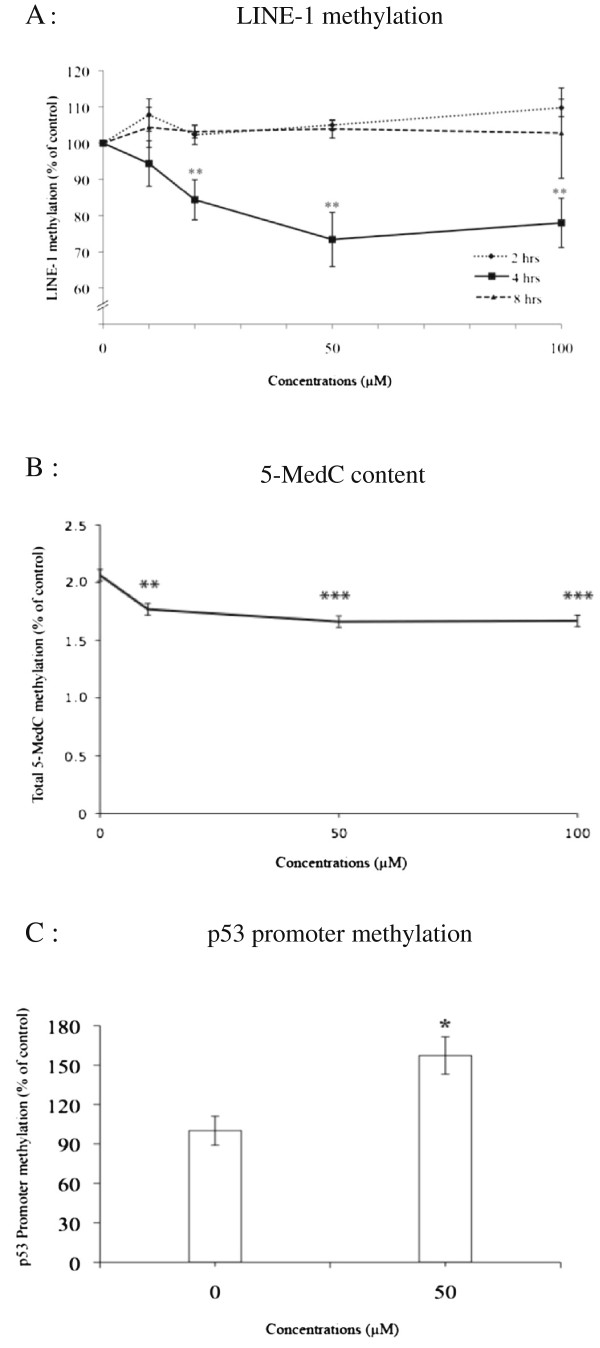
**Lymphoblasts were treated with NaAsO**_2_**at 0, 10, 20, 50 and 100 μM for two, four or eight hours.** Levels of DNA methylation expressed as % of controls were illustrated as a dose-response and time-course study on global LINE-1 methylation determined by COBRA and quantified by capillary gel electrophoresis using a Bioanalyzer **(A)**, dose-response of arsenite treatment on total 5-MedC content determined by HPLC-MS/MS in lymphoblasts at four hours of treatment **(B)** and p53 promoter methylation in lymphoblasts treated with arsenite at 50 μM for four hours **(C)**. Each point represents the mean ± SE from three independent experiments. *,** and *** represent statistically significant difference from control at p < 0.05, 0.01 and p < 0.001, respectively.

a) Short-term exposure to arsenic *in vitro* was conducted by treatment of human lymphoblasts with sodium arsenite at concentrations ranging from 10-100 μM for two, four and eight hours. A significant reduction of LINE-1 methylation was observed only for four hours of treatment. A progressive decrease in methylation level was observed between 10 to 50 μM, then levelling off towards 100 μM. Global DNA methylation levels in lymphoblasts-treated with arsenite for four hours was 93.54 %, 83.68 %, 62.55 %, and 66.16 % of the control levels at 10, 20, 50 and 100 μM, respectively. These decreases were statistically significant at 20 μM (p < 0.05), 50 μM (p < 0.01) and 100 μM (p < 0.01). A maximum reduction of methylation level was observed at 50 μM (Figure 2A).

From this observation, it was decided that the optimum period for arsenite exposure in the subsequent experiments would be four hours. Measurement of 5-MedC content in lymphoblasts exposed to different concentrations of arsenite from 10-100 μM showed that levels were significantly decreased at all concentrations tested (p < 0.01 at 10 μM, p < 0.001 at 50 and 100 μM). The lowest methylation level was observed at concentration of 50 μM (Figure 2B). The profile of 5MedC content in response to arsenite exposure was consistent with the decrease in LINE-1 methylation.

We then further explored the effect on a specific gene, p53, at the promoter region. Using methylation-specific restriction endonuclease digestion assay and Bioanalyzer quantification, p53 promoter methylation was found to increase after arsenite treatment. In arsenite-treated lymphoblasts (50 μM for four hours), the promoter region of p53 was hypermethylated by approximately 1.5-fold compared to the controls (p < 0.05) (Figure 2C).

**Figure 3:**
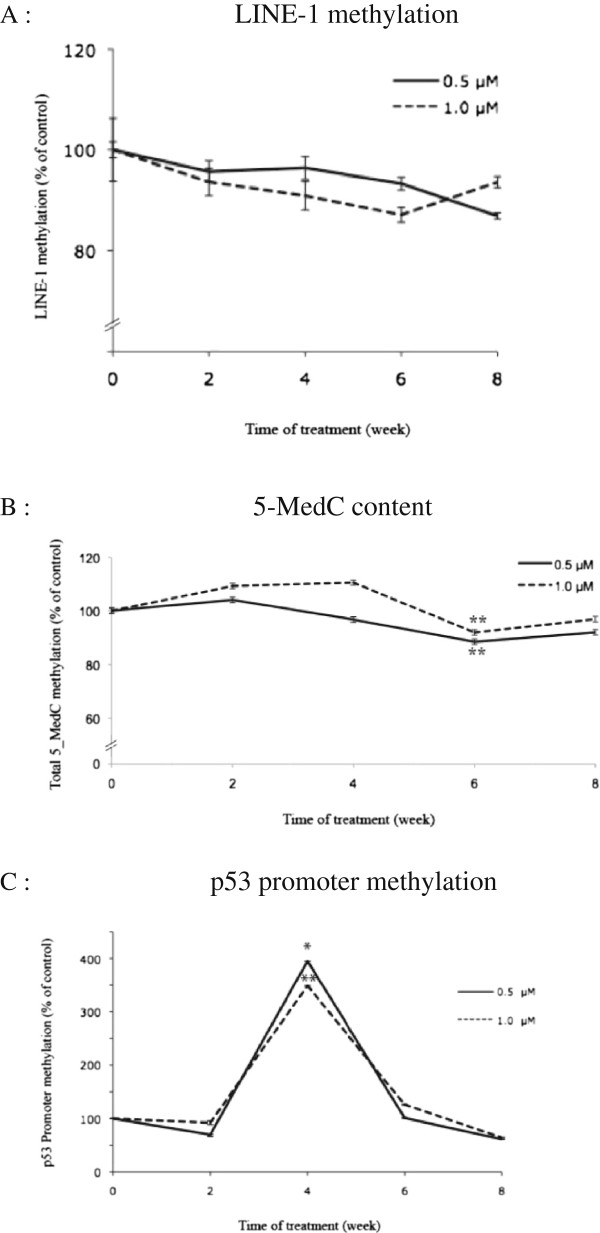
**Lymphoblasts were treated with NaAsO**_2_**(0.0, 0.5 and 1.0 μM) for two up to eight weeks.** The experiment was performed twice. Dose-response and time-course of arsenite treatment on levels of DNA methylation expressed as % of controls was determined as Global LINE-1 methylation determined by COBRA, quantified by capillary gel electrophoresis using a Bioanalyzer **(A)**, total 5-MedC content determined by HPLC-MS/MS **(B)** and p53 promoter methylation **(C)**. Each point represents the mean ± SE from three independent experiments. * and ** represent statistically significant difference from control at p < 0.05 and p < 0.01, respectively.

b) Long-term exposure to arsenite for up to eight weeks in the lymphoblast cells was carried out at concentrations of 0.5 and 1 μM. LINE-1 methylation in treated cells gradually decreased starting at two weeks and over the remaining six weeks of treatment. The level of LINE-1 methylation in arsenite-treated cells at 1.0 μM was slightly lower than that at 0.5 μM at all time points, except at eight weeks of treatment (Figure 3A).

The level of total 5-MedC content in the treated lymphoblast cell line was not significantly different from the untreated levels except at six weeks of treatment, which significantly reduced (p < 0.01) at six weeks of treatment, both at 0.5 and 1.0 μM. The lowest levels of 5-MedC were 88 % and 91 % of the control levels at 0.5 and 1.0 μM, respectively (Figure 3B).

Two weeks of treatment with arsenite showed no effect on p53 promoter methylation in the lymphoblast cell line, but a dramatic increase (approximately 300 % of the control level) was observed at four weeks (p < 0.05 and p < 0.01 at arsenite concentrations of 0.5 and 1.0 μM, respectively), with a subsequent decrease to control levels by six weeks. Similar profiles of p53 promoter methylation alterations in lymphoblasts with respect to time course of treatment were observed for 0.5 and 1.0 μM of arsenite (Figure 3C).

## **Discussion**

Gestation period is a period of high sensitivity to chemical exposure, since it covers the whole period of fetal development. There is accumulating evidence that *in utero* exposure to arsenic is associated with congenital defects and long-term disease consequences, such as cancer. A recent finding in whole life arsenic exposure in an animal model indicated that *in utero* arsenic exposure may dictate target site of tumor while other periods of exposure could act to enhance the carcinogenic response [38]. Transplacental exposure to arsenic is thought to lead to the gestational disruption of epigenetic gene imprinting during this susceptible period of fetal development [25]. Aberrant DNA methylation and gene expression in livers were observed in newborn mice transplacentally exposed to inorganic arsenic [39].

In human, *in utero* or early childhood exposure to arsenic in drinking water was associated with increased mortality in young adults in Chile from both malignant and non-malignant lung disease [18,19]. Our previous report demonstrated for the first time that arsenic exposure *in utero* resulted in gene expression changes in the newborns of mothers living in arsenic contaminated areas during pregnancy [28]. The present study further investigates other effects of such exposure such as those on DNA methylation, which plays a key role in the control of gene expression.

Globally, the most important source of arsenic exposure in humans is drinking water [40]. This study clearly showed that arsenic contamination in various sources of consumable water was much higher in the contaminated site, with arsenic concentrations in drinking water being approximately 46-fold higher than those from the control site (8.38 *versus* 0.18 μg/L, p < 0.05). Although the level of arsenic detectable in drinking water was below the established WHO standard of 10 μg/L, arsenic exposure was found to be significantly higher in the newborns of mothers living in this contaminated site. Arsenic exposure in these newborns was assessed using biomarkers of exposure, including arsenic concentrations in biological samples, i.e cord blood, nails and hair. Detection of arsenic in these biological samples is indicative of systemic absorption after arsenic exposure [41]. When compared to the control group, exposed newborns had significantly higher concentrations of arsenic in cord blood (3-fold), nails (10- and 20-fold in toenails and fingernails, respectively) and hair (5-fold). Based on the fold level of increase, arsenic accumulation in hair and nails could be considered good biomarkers of arsenic exposure. Absorbed arsenic accumulates in hair and nails, and it is thought that this involves the binding of As^iii^ to the sulhydryl groups in keratin. Due to the slow growth of hair and nails, arsenic accumulation is an indication of past arsenic exposure and thus can be used as biomarkers for long-term arsenic exposure. A significant correlation has been reported between arsenic concentrations in drinking water and levels of accumulation in nails [42] and hair [43]. Analysis of arsenic in blood is suitable for recent and high level of exposure, but it may not be a reliable biomarker of arsenic exposure because it is cleared rapidly, particularly for low levels of inorganic arsenic [3,44].

Growing evidence indicates that arsenic carcinogenesis involves, either directly or indirectly, altered epigenetic regulation in gene expression changes induced by arsenic exposure [45]. Epigenetic changes in methylation patterns are increasingly implicated in cancer development [10]. The mechanism underlying the epigenetic changes in DNA methylation mediated by arsenic has been proposed to involve arsenic modulating the activity of DNA methyltransferase (DNMT), which catalyzes the transfer of a methyl group from S-adenosylmethionine (SAM) on to the 5’-position of cytosine at a CpG dinucleotide to produce 5-methylcytosine. It has been reported that arsenic inhibits DNMT activity and expression of *DNMT1DNMT3A*, and *DNMT3B*[46-48] as well as depletes SAM, resulting in genome-wide hypomethylation [7,48]. In addition, it has been reported that arsenic mediates the disruption of normal epigenetic control at specific loci, such as the promoter regions of the tumor suppressor genes *p53* and *p16*, which may result in aberrant gene expression and cancer [39,49]. Generally, global hypomethylation, as well as hypermethylation, have been associated with reduced chromosomal stability and altered genome function. Gene promoter hypermethylation is associated with decreased gene expression [50].

In this study, impacts of arsenic exposure on DNA methylation were investigated, focusing on global and gene-specific methylation. Methylation of repetitive elements of LINE-1 represents approximately 17 % of the human genome [51]. Because of high level of methylation in normal tissue and close correlation with genomic DNA methylation content, LINE-1 methylation status has been used as a surrogate marker for estimating the genome-wide methylation level [34].

The present study showed that prenatal arsenic exposure did not cause a significant change in global LINE-1 methylation in newborns from the arsenic-exposed mothers, compared to those from the unexposed mothers living in the control site. This observation is consistent with those from animal studies which *in utero* arsenic exposure in mice was not associated with any changes in global DNA methylation [23,39].

To achieve a better understanding of the epigenetic effects of arsenic exposure, DNA methylation was further investigated *in vitro*, through short-term and long-term exposure using a human lymphoblast cell line. The low concentrations of arsenic used in the long-term *in vitro* study are non-cytotoxic, corresponding to 37 and 74 ppb, which are level that can be found in contaminated environment, since 0.67 μM of arsenite is equivalent to 50 ppb [52]. The short-term *in vitro* study used higher concentrations of arsenic, including 10, 20, 50 and 100 μM, equivalent to 750, 1500, 3700 and 7400 ppb, respectively, to demonstrate more observable effects of arsenic exposure and to better characterize of its mechanism of action. These high concentrations of arsenic in water have been found in some areas, such as Taiwan (above 600 μg/L) [53] and up to 7500 μg/L in Argentina [54].

Apart from LINE-1 methylation, total 5-MedC content was also determined as another indicator of genome-wide methylation. Measurement of 5-MedC content using HPLC-MS/MS has been shown to be a rapid, sensitive and specific technique for detection of global methylation in the genome [55,56]. Following short-term *in vitro* arsenite treatment, the dose-response and time-course study clearly demonstrated a significant global hypomethylation, observed as a reduction in LINE-1 methylation and total 5-MedC content in lymphoblasts exposed to arsenite (10-100 μM) for four hours, but not at two hours or eight hours. The reduction of LINE-1 methylation observed at four hours was found to correlate significantly with the highest levels of DNA strand breaks in arsenite-treated lymphoblasts (data not shown), suggesting a possible link between DNA damage (which, in the case of arsenic, is known to be due to oxidative stress) and LINE-1 methylation. This observation was consistent with the previous finding that high levels of oxidative DNA damage may induce global DNA hypomethylation as a result of oxidative damage of methylated cytosine residues, which is a template recognition site for DNMT [57]. In the long-term study, global DNA hypomethylation was also observed following treatment with low concentrations of arsenite at 0.5 and 1.0 μM. These results suggested that global DNA hypomethylation can be mediated by arsenic exposure at low concentrations, but the extent of methylation is dependent on the concentration and duration of exposure. Global DNA hypomethylation has been proposed to contribute to carcinogenesis *via* activation of oncogenes and inactivation of tumor suppressor genes, as well as chromosomal aberrations and increased mutation rates [58-60]. As a consequence, arsenic exposure may lead to diseases and cancer outcomes through genome-wide hypomethylation.

Regarding gene-specific methylation, methylation at the promoter region of p53 was investigated since p53 is a key factor in the damage-signaling pathway. The tumor suppressor *p53* gene plays a significant role in a variety of cellular functions, including cellular stress, regulation of cell cycle, apoptosis and DNA repair. Available evidence indicates that arsenic exposure predisposes cells to malignant transformation *via* alterations of *p53* expression and function [61], *p53* mutation [62], as well as alterations of p53 methylation [14].

In this study, global DNA methylation levels were not significantly changed, while p53 promoter methylation was slightly increased in exposed newborns. We observed high variation in arsenic exposure levels within the exposed group, which led to a broad range of p53 promoter methylation levels. However, the significant correlation between p53 promoter methylation and accumulation of arsenic in nail samples indicated that arsenic exposure increased p53 promoter methylation. These results appear to be in line with observations in the animal study that arsenic exposure *in utero* may affect specific DNA methylation changes at targeted sites rather than global hypomethylation [7]. A slight increase in p53 promoter methylation as well as unchanged global methylation in these arsenic-exposed newborns was possibly due to a relatively low level of arsenic exposure in our study. Level of arsenic contamination in the environment and particularly in drinking water in this study site (<10 μg/L) was lower than in other arsenic-contaminated sites. Arsenic levels in drinking water were greater than 400 μg/L in West Bengal, India [63] and approximately 50 μg/L in Bangladesh [64].

Short-term *in vitro* arsenite treatment clearly resulted in hypermethylation of p53 at the promoter region in treated-lymphoblasts at 50 μM. This result is in agreement with previous studies in which hypermethylation of p53 was observed in arsenite-treated A549 cells [14], as well as in arsenic exposed patients with skin cancer who were chronically exposed to high arsenic-contaminated drinking water at levels ranging between 300-1000 μg/L [15]. Hypermethylation of p53 promoter region may be due to overexpression of *DNMT1*, which was shown to be overexpressed and correlated with hypermethylation of CpG island of other genes, i.e *hMLH1 * and *THBS1 *[65]. This may suggest the possible involvement of increased expression of *DNMT1* in hypermethylation of specific-genes. However, long-term *in vitro* arsenite treatment showed a transient hypermethylation of p53 in lymphoblasts at six weeks of treatment, then decreased. This may be due to the fact that prolonged arsenic exposure may cause depletion of the SAM pool through overconsumption of methyl groups by DNMT, resulting in an inability to maintain methylated cytosine in DNA. In addition, evidence from the *in vitro* study showed that DNA methylation status in specific genes may change rapidly in response to environmental stressors [66].

## **Conclusions**

Our results demonstrate that in humans, *in utero* exposure to arsenic in the environment affects DNA methylation, particularly at the promoter region of p53 causing hypermethylation although alterations in global DNA methylation were not detectable. A significant correlation between p53 promoter hypermethylation and arsenic accumulation in nails was observed. These findings confirmed by *in vitro* exposure studies in human lymphoblasts that arsenic causes hypermethylation of p53 promoter region as well as global hypomethylation. These results contribute possible mechanistic evidence for the previously reported epidemiological observation that *in utero* exposure to arsenic in drinking water was associated with an increased mortality in young adults in Chile from malignant and non-malignant lung diseases.

## **Abbrevations**

5-MedC, 5-methyl-2'-deoxycytidine; COBRA, Combined bisulfite restriction analysis; FBS, Fetal bovine serum; ICP-MS, Inductively coupled plasma mass spectrometry; HPLC-ESI-MS/MS, High pressor liquid chromatography-electrospray ionization-tandem mass spectrometry; LINE-1, Long interspersed nuclear element-1.

## **Competing interests**

No competing interests.

## **Authors’ contributions**

PI and SW performed analysis of cord blood samples and *in vitro* experiments. PN coordinated laboratory experiments and field studies, and prepared the manuscript. KC performed analysis of arsenic in the environmental and biological samples. WAS advised PI in technical aspects. CM coordinated the overall project implementation, particularly at the study sites. MR as principal investigator, conceived and designed the whole study and experiments, sought funding support as well as wrote the manuscript. All authors read and approved the final manuscript.
